# MVHGCN: Predicting circRNA-disease associations with multi-view heterogeneous graph convolutional neural networks

**DOI:** 10.1371/journal.pcbi.1013225

**Published:** 2025-06-19

**Authors:** Yan Miao, Xuan Tang, Chunyu Wang, Zhenyuan Sun, Guohua Wang, Shan Huang

**Affiliations:** 1 College of Computer and Control Engineering, Northeast Forestry University, Harbin, China; 2 Faculty of Computing, Harbin Institute of Technology, Harbin, China; 3 Department of Neurology, Harbin Medical University, Harbin, China; University of Electronic Science and Technology, CHINA

## Abstract

Circular RNA, a class of RNA molecules gaining widespread attentions, has been widely recognized as a potential biomarker for many diseases. In recent years, significant progress has been made in the study of the associations between circRNA and diseases. However, traditional experimental methods are often inefficient and costly, making computational models an effective alternative. Nevertheless, existing computational methods still face challenges such as data sparsity and the difficulty of confirming negative samples, which limits the accuracy of predictions. To address these challenges, a novel computational method, namely MVHGCN, is proposed based on multi-view and graph convolutional networks to predict potential associations between circRNA and diseases. MVHGCN first constructs a heterogeneous graph and generates feature descriptors by integrating multiple databases. Then it extracts different connection views of circRNA and diseases through meta-paths, maximizing the utilization of known association information, and aggregates deep feature information through graph convolutional networks. Finally, a MLP is used to predict the association scores. The experimental results show that MVHGCN significantly outperforms existing methods on benchmark datasets by 5-fold cross-validation. This research provides an effective new approach to studying the associations between circRNAs and diseases, capable of alleviating the problem of data sparsity and accurately identifying potential associations.

## Author summary

Circular RNA has garnered significant attention due to its unique structure and potential role in regulating gene expression. It can interact with microRNAs, preventing the degradation of messenger RNAs, and influencing a network of competing RNAs. However, traditional experimental methods are often inefficient and costly, making computational models an effective alternative. Nevertheless, existing computational methods still face challenges such as data sparsity and the difficulty of confirming negative samples, which limits the accuracy of predictions. To address these issues, I propose a novel method called MVHGCN, which leverages heterogeneous graphs and graph convolutional networks to predict associations between circRNAs and diseases. This approach integrates diverse data views and uses deep learning to provide accurate predictions. When compared to existing methods, MVHGCN significantly outperforms them, demonstrating its potential to advance disease research and the development of therapeutic targets. The results underscore the importance of improving prediction models in the study of circRNA-disease relationships, ultimately contributing to more effective disease diagnosis and treatment strategies.

## Introduction

Circular RNA (circRNA) is a type of RNA molecule that exists widely in various biological categories [1], which has received widespread attention in recent years. Existing studies have demonstrated that once the circRNA is transported to the cytoplasm, it can act as a microRNA (miRNA) sponge [2]. By competitive binding to miRNAs [3], circRNA inhibits miRNA-mediated degradation of messenger RNA (mRNA) targets [4], thus regulating the function of the competing endogenous RNA network (ceRNA) [5]. Compared to linear non-coding RNAs (lncRNA), circRNA is characterized by a covalently closed circular structure. This specific structure endows circRNA with greater stability in vivo compared to lncRNA, conferring enhanced resistance to nucleases and exoribonucleases [6], potentially making circRNA a valuable biomarker for diseases [7]. Consequently, the discovery of new associations between circRNAs and diseases can provide crucial insights for disease researches and the development of drug targets [8].

Several tools have been proposed to classify circRNA-disease associations [9], which can be broadly categorized into three types: network-based methods, machine learning-based methods, and deep learning-based methods. Network-based methods predict associations by constructing a circRNA-disease association network before the progress of random walk and message propagation. For instance, KATZHCDA [10] employed a heterogeneous network constructed by circular RNA expression profiles, disease phenotype similarity, and Gaussian interaction profile kernel similarity, with KATZ algorithm to predict associations. RWRKNN [11] integrated the restart random walk algorithm with the k-Nearest Neighbor (KNN) algorithm to predict the associations between circRNAs and diseases. Machine learning-based methods construct circRNA and disease features to enrich association information and then use machine learning algorithms to predict these associations. iCircDA-MF [12] performed non-negative matrix factorization on the association matrix to compute association scores. NMFCDA [13] predicted associations between circRNAs and diseases by integrating randomized neural network pseudoinverse learning with non-negative matrix factorization. Deep learning-based methods employs deep learning techniques to extract latent features for predicting associations [14]. Lan *et al*. [15] employed a graph attention network (GAT), which aggregates information through multiple layers of propagation, followed by a Multi-Layer Perceptron (MLP) for prediction. GMNN2CD [16] integrated graph autoencoder and variational inference within a graph Markov neural network framework, leveraging feature inference (GNNq) and label propagation (GNNp) trained alternately via a variational EM algorithm to predict circRNA–disease associations. Wu *et al*. [17] proposed a method based on Transformer for knowledge representation learning and attention propagation layers to obtain high-quality embeddings, followed by a MLP for predicting associations. MSMCDA [18], a method that integrates shared units and attention mechanisms to fuse similarity and meta-path networks for circRNA–disease association prediction, enhanced by contrastive learning and followed by an MLP classifier.

Although the aforementioned methods have demonstrated excellent performance, there are still two general issues: (1) Current databases contain a relatively limited number of circRNA-disease associations, leading to a data sparsity problem that severely restricts the predicting accuracy. (2) Verified circRNA-disease non-associations are typically difficult to obtain, making it challenging to determine negative samples.

To address these two problems, a novel computational method, namely MVHGCN, is proposed to predict circRNA-disease associations. It consists of four main components. Firstly, MVHGCN computes feature descriptors of circRNA and disease respectively from known circRNA-disease associations. Secondly, it acquires association views with different connectivity patterns from a heterogeneous graph through meta-paths. Then, these diverse connectivity views are fused, and deep information is aggregated through a graph convolutional network to obtain the final representations. Finally, a MLP is employed to obtain the association scores between circRNAs and diseases. To verify the effectiveness of MVHGCN, it is compared to six benchmark methods, including GMNN2CD [16], MLNGCF [19], AE-RF [20], CircWalk [21], KGETCDA [17], and KGRACDA [22]. The experimental results demonstrate that MVHGCN outperforms these methods. Specifically, for the key metric AUC, taking Dataset 1 as an example, MVHGCN achieves the highest value of 99.3%, which is 4.6% higher than GMNN2CD, 29.3% higher than MLNGCF, 19.2% higher than AE-RF, 13.8% higher than CircWalk, 33.2% higher than KGETCDA, and 12.2% higher than KGRACDA, respectively.

## Materials and methods

### The framework of MVHGCN

**Fig 1:**
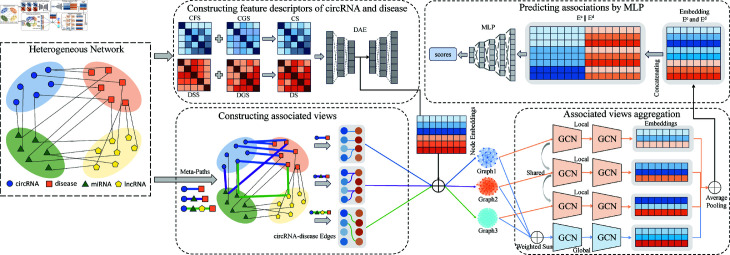
The overall workflow of MVHGCN.

The overall workflow of MVHGCN is shown in Fig 1. Initially, multi-source data are integrated to construct a large-scale heterogeneous graph, from which a circRNA-disease association matrix is obtained. Subsequently, four feature matrices, the disease semantic similarity matrix (DSS), the disease GIP kernel similarity matrix (DGS), the circRNA functional similarity matrix (CFS), and the circRNA GIP kernel similarity matrix (CGS), are computed from the circRNA-disease association matrix. These matrices are then fused to obtain the feature descriptor of circRNA and disease. Next, in the heterogeneous graph, different association views are acquired based on the distinct connection patterns between circRNAs and diseases using meta-paths. Thereafter, the association views are aggregated using global and local view aggregation to obtain the final representation. Finally, the prediction layer of an MLP is employed to output association scores.

### Constructing feature descriptors of circRNA and disease

To extract high-quality initial features accurately, four matrices (DSS, DGS, CFS, and CGS) were calculated from the circRNA-disease association matrix. These similarity matrices were integrated into a feature descriptor for subsequent analysis.

#### Disease semantic similarity (DSS)

Based on existing research [23], disease semantic similarity is calculated using Medical Subject Headings (MeSH), which are provided by the National Center for Biotechnology Information (NCBI). In the MeSH database, the association information between diseases is represented by a Directed Acyclic Graph (DAG) denoted as $DAG_{d}=(d,A_{d},R_{d})$, where node *d* represents a disease, *A*_*d*_ is the set of ancestor nodes of node *d* (including node *d* itself), and *R*_*d*_ is the set of relationships associated with node *d*. DSS was calculated by two methods. In the first method, the contribution of disease *j* to disease *i* was calculated by:

$$D_{i}(j)=\left\{ \begin{array}{cc} 1 & \text{if }j=i\\ \max\{\lambda \cdot D_{i}(j^{\prime })|j^{\prime }\in \text{children of }j\} & \text{if }j\neq i \end{array}\right.$$ (1)

where λ represents the semantic contribution factor between diseases, which is typically set to 0.5. The semantic value *DV* for disease *i* is calculated by:

$$DV(i)=\sum_{j\in A_{d}}D_{i}(j)$$ (2)

In DAG, the greater the number of nodes shared between diseases, the higher their similarity. The semantic similarity between disease *m* and disease *n*, denoted as DSS1(d(m,d(n)), is calculated as:

$$DSS_{1}(d(m),d(n))=\frac{\sum_{d\in A_{d(m)}\cap A_{d(n)}}D_{d(m)}(j)+D_{d(n)}(j)}{DV(d(m))+DV(d(n))}$$ (3)

The second method posits that the number of diseases plays a crucial role in disease contribution, and diseases that appear fewer times in the DAG may be more significant. It is calculated as:

$$D_{i}^{\prime }(j)=-\log\left(\frac{\text{num}(DAG_{s}(j))}{\text{num}(diseases)}\right)$$ (4)

where *num*(*DAG*_*s*_(*j*)) is the number of occurrences of disease j in its associated subgraph, and *num*(*diseases*) is the total number of diseases in DAGs. Then, the semantic similarity between disease *m* and disease *n*, denoted as DSS2(d(m),d(n)), is calculated as:

$$DSS_{2}(d(m),d(n))=\frac{\sum_{j\in A_{d(m)}\cap A_{d(n)}}D_{d(m)}^{\prime }(j)+D_{d(n)}^{\prime }(j)}{DV^{\prime }(d(m))+DV^{\prime }(d(n))}$$ (5)

$$DV^{\prime }(i)=\sum_{j\in A_{d}}D_{i}^{\prime }(j)$$ (6)

Ultimately, $DSS=\frac{DSS_{1}+DSS_{2}}{2}$.

#### Disease GIP kernel similarity (DGS)

Due to the sparsity of disease semantic similarity data, not all diseases have semantic similarity information, which limits the comprehensive representation of disease features. We additionally employes the Gaussian Interaction Profile Kernel Similarity (GIPKS) [16] is additionally employed to compute disease similarities to obtain more comprehensive information. The GIP kernel similarity between disease *i* and disease *j* is calculated as:

$$DGS(d_{i},d_{j})=\exp\left(-\delta {\left\|A(d_{i})-A(d_{j})\right\|}^{2}\right)$$ (7)

$$\delta =\frac{1}{\frac{1}{n_{d}}\sum_{i=1}^{n_{d}}{\left\|A(d_{i})\right\|}^{2}}$$ (8)

where δ represents the bandwidth number, *n*_*d*_ denotes the total number of diseases, and *A*(*d*_*i*_) and *A*(*d*_*j*_) are corresponded to the *i*-th and *j*-th columns of the circRNA-disease association matrix, respectively.

#### CircRNA functional similarity (CFS)

Based on the hypothesis that similar diseases have similar circRNAs [17], the CFS can be calculated as:

$$CFS(c_{i},c_{j})=\frac{\sum_{1\leq m\leq |D_{i}|}S(d_{m},D_{j})+\sum_{1\leq n\leq |D_{j}|}S(d_{n},D_{i})}{\left|D_{i}|+|D_{j}\right|}$$ (9)

$$S(d_{m},D_{j})=\max_{1\leq s\leq |D_{j}|}\left(DS(d_{m},d_{s})\right)$$ (10)

where *c*_*i*_ and *c*_*j*_ represent the *i*-th and *j*-th circRNA, respectively, and *D*_*i*_ and *D*_*j*_ denote the sets of diseases associated with these two circRNA.

#### CircRNA GIP kernel similarity (CGS)

Similar to DGS, the CGS can be calculated as:

$$CGS(c_{i},c_{j})=\exp\left(-\delta {\left\|A(c_{i})-A(c_{j})\right\|}^{2}\right)$$ (11)

$$\delta =\frac{1}{\frac{1}{n_{c}}\sum_{i=1}^{n_{c}}{\left\|A(c_{i})\right\|}^{2}}$$ (12)

where δ represents the number of bandwidths, *n*_*c*_ denotes the total number of diseases, and *A*(*c*_*i*_) and *A*(*c*_*j*_) respectively indicate the *i*-th and *j*-th rows of the circRNA-disease association matrix.

#### Constructing feature descriptors

If there exists a DSS between two diseases, the disease similarity DS(d(i),d(j)) is defined as the DSS between these two diseases. If there is no semantic similarity, it is defined as their DGS, which is calculated as:

$$DS(d(i),d(j))=\left\{ \begin{array}{cc} DSS(d(i),d(j)) & \text{if }DSS(d(i),d(j))\neq 0\\ DGS(d(i),d(j)) & \text{if }DSS(d(i),d(j))=0 \end{array}\right.$$ (13)

The definition of circRNA similarity is similar to that of disease similarity. If two circRNAs exhibit functional similarity, their circRNA similarity CS(c(i),c(j)) is defined as the CFS between them. If there is no functional similarity, it is defined as their CGS, which is calculated as:

$$CS(c(i),c(j))=\left\{ \begin{array}{cc} CFS(c(i),c(j)) & \text{if }CFS(c(i),c(j))\neq 0\\ CGS(c(i),c(j)) & \text{if }CFS(c(i),c(j))=0 \end{array}\right.$$ (14)

Deep Autoencoder [24] is an unsupervised learning model based on neural networks, commonly used for tasks such as dimensionality reduction, feature extraction, and data denoising. After calculating the disease similarity and circRNA similarity, a deep autoencoder is utilized to unify their dimensionality to obtain initial feature descriptors for diseases and circRNAs, respectively.

### Constructing associated views

Meta-paths [25] are generally used to describe the relationships between nodes of different types through which potential information among different nodes in heterogeneous graphs can be mined. A meta-path *P* is defined as a path $A_{1}\overset{R_{1}}{\to }A_{2}\overset{R_{2}}{\to }\cdots \overset{R_{l}}{\to }A_{l+1}$, abbreviated as $A_{1}A_{2}\ldots A_{l+1}$, which describes the composite relationship $R=R_{1}\circ R_{2}\circ \cdots \circ R_{l}$ between node types *A*_1_ and *A*_*l* + 1_. *A* and *R* represent the sets of node and edge types, respectively, in the heterogeneous graph, and ∘ denotes the composition operator for relationships.

To accurately obtain the association information between circRNAs and diseases, meta-paths are utilized to extract various connections between circRNAs and diseases from the heterogeneous graphs. These meta-paths are used to construct different association views within the heterogeneous graph, allowing for a multi-view analysis of the interactions between circRNAs and diseases. Each association view is a bipartite graph that contains only circRNAs and diseases, and simultaneously extracts the structural information of the heterogeneous graph. For MVHGCN, 10 meta-paths are selected to analyze the association relationships, which are detailed as:


$$\begin{array}{c} P_{\text{cd}}=\text{circRNA-disease}\\ P_{\text{cmd}}=\text{circRNA-miRNA-disease}\\ P_{\text{cdcd}}=\text{circRNA-disease-circRNA-disease}\\ P_{\text{cdmd}}=\text{circRNA-disease-miRNA-disease}\\ P_{\text{cmcd}}=\text{circRNA-miRNA-circRNA-disease}\\ P_{\text{cdld}}=\text{circRNA-disease-lncRNA-disease}\\ P_{\text{cmld}}=\text{circRNA-miRNA-lncRNA-disease}\\ P_{\text{cmlmd}}=\text{circRNA-miRNA-lncRNA-miRNA-disease}\\ P_{\text{cdmld}}=\text{circRNA-disease-miRNA-lncRNA-disease}\\ P_{\text{cdlmd}}=\text{circRNA-disease-lncRNA-miRNA-disease} \end{array}$$

Given the complexity of the constructed heterogeneous graph structure and the sharp increase in the number of associations between circRNAs and diseases as the length of a meta-path increases, DPRel [26], a meta-path-based relevance measurement method specifically designed for heterogeneous networks, is used in MVHGCN to calculate the association scores between circRNAs and diseases. This approach effectively filters out circRNA-disease pairs with low association scores, thereby enhancing the accuracy and effectiveness of the association analysis. Traditional methods with homogeneous networks often fail to preserve diverse semantic information. However, DPRel not only retains such information but also effectively computes the relevance between objects along the paths, so that it is applicable to relevance measurement between nodes of the same type and different types. Specifically, given a meta-path $P=(A_{1}A_{2}\ldots A_{l+1})$, the DPRel relevance between the source object $a_{1i}\in A_{1}$ and the target object $b_{(l+1)j}\in A_{l+1}$ was defined as:

$$DPRel(a_{1}i,b_{(l+1)j}|P)=\frac{w(a_{1i},b_{(l+1)j})\left(\frac{1}{\text{deg}(a_{1i})}+\frac{1}{\text{deg}(b_{(l+1)j})}\right)}{\frac{1}{\text{deg}(a_{1i})}\sum_{j}w(a_{1i},b_{(l+1)j})+\frac{1}{\text{deg}(b_{(l+1)j})}\sum_{i}w(a_{1i},b_{(l+1)j})}$$ (15)

where $w(a_{1i},b_{(l+1)j})$ represents the number of paths connecting *a*_*li*_ and *b*_(*i* + 1)*j*_, *deg*(*a*_*li*_) and *deg*(*b*_(*i* + 1)*j*_) are the degrees of nodes *a*_*li*_ and *b*_(*i* + 1)*j*_ in the heterogeneous graph, respectively.

### Associated views aggregation

To embed high-order features from multiple correlated views into node embeddings, an Associated View Aggregation Mechanism (AVAM) is constructed. It consists of two key components: Global View Aggregation (GVA) and Local View Aggregation (LVA). The GVA is built on the deep aggregation mechanism [27]. LVA is established to focus on the information aggregation among nodes within each view, further enhancing the richness of learned embeddings.

#### Global View Aggregation (GVA)

As a composite view of multiple perspectives, GVA reveals different correlation patterns at a deeper level. By adaptively learning the importance of each correlation view, GVA achieves better performance on information aggregation. Based on the generated correlation views, GVA distinguishes their importance and weights the correlation views by a set of learnable weighting parameters α:

$$A_{\text{global}}=\sum_{i=1}^{N}\alpha_{i}A_{i}$$ (16)

where *N* represents the number of associated views, and $A_{i}\in {\mathbb{R}}^{n\times n}$ is the adjacency matrix of the *i*-th associated views. Subsequently, the aggregated matrix is input into a simplified graph convolutional network for convolution operations, during which no nonlinear activation functions are employed:

$$E_{\text{global}}^{\left(1\right)}=A_{\text{global}}\cdot X\cdot W_{\text{global}}^{\left(1\right)},$$ (17)

where $X\in {\mathbb{R}}^{n\times m}$ represents the node embedding matrix, and $W_{\text{global}}^{\left(1\right)}\in {\mathbb{R}}^{m\times m}$ denotes a learnable weight matrix. Consequently, a single-layer GCN [28] is used to effectively learn node representations that incorporate interaction information from all associated views. To capture deeper relational information, this concept is extended to *l* layers:

$$E_{\text{global}}^{\left(l\right)}=A_{\text{global}}\cdot E_{\text{global}}^{\left(l-1\right)}\cdot W_{\text{global}}^{\left(l\right)}=A_{\text{global}}^{l}\cdot X\cdot \underset{l}{\underbrace{W_{\text{global}}^{\left(1\right)}\cdots W_{\text{global}}^{\left(l\right)}}}$$ (18)

Considering the over-smoothing problem in graph neural networks, only two convolutional layers are used in MVHGCN. To fully capture all interaction information across various depth-related views, the outputs of each layer are fused to obtain the final node representations:

$$E_{\text{global}}=\frac{1}{l}\sum_{i=1}^{l}E_{\text{global}}^{\left(i\right)}\in {\mathbb{R}}^{n\times m}$$ (19)

#### Local View Aggregation (LVA)

LVA learns the importance of each associated view separately to generate respective node representations, so that it could focus on their inner information among nodes within each view. Specifically, every view is input to a simplified graph convoluational network for convolution operations:

$$E_{\text{i}}^{\left(l\right)}=A_{\text{i}}\cdot E_{\text{i}}^{\left(l-1\right)}\cdot W_{\text{i}}^{\left(l\right)}=A_{\text{i}}^{l}\cdot X\cdot \underset{l}{\underbrace{W_{\text{i}}^{\left(1\right)}\cdots W_{\text{i}}^{\left(l\right)}}}$$ (20)

where $E_{i}\in {\mathbb{R}}^{n\times m}$ represents the node embedding matrix for the *i*-th view, and $W_{i}\in {\mathbb{R}}^{m\times m}$ denotes the learnable weight matrix for the *i*-th view. Additionally, all parameters in the GCN are shared across all views.

The final node representations are obtained by averaging the last layer outputs of each view with *E*_*global*_, as shown below:

$$E^{ci}=\frac{1}{N+1}\left(\sum_{m=1}^{N}E_{\text{m}}^{ci}+E_{\text{global}}^{ci}\right)$$ (21)

$$E^{dj}=\frac{1}{N+1}\left(\sum_{m=1}^{N}E_{\text{m}}^{dj}+E_{\text{global}}^{dj}\right)$$ (22)

where $E_{\text{m}}^{ci}$ represents the embedding of the *i*–th circRNA in the *m*-th view.

### Predicting associations by MLP

Based on the aforementioned process, the final representations of circRNAs and diseases are obtained by connecting *E*^*ci*^ and *E*^*dj*^ as the embedding representations for *circRNA ci* and disease *dj*, before being input to an MLP to obtain their association scores:


$$S_{ij}=\sigma_{l}\left(W_{l}\left(\sigma_{l-1}\left(\cdots \sigma_{1}\left(W_{1}\left(E^{ij}\right)+b_{1}\right)\cdots \right)+b_{l-1}\right)+b_{l}\right)\;E^{ij}=E^{ci}∥E^{dj}$$ (23)

where σ, *W*, and *b* represent the activation function, learnable weight matrix, and bias, respectively. ReLU is chosen as the activation function for hidden layers, and the sigmoid function is chosen for the output layer.

Contrastive learning has demonstrated its superiority in various graph learning tasks. Therefore,InfoNCE [29] (Information Noise Contrastive Estimation) is a loss function specifically designed for contrastive learning tasks. Its primary goal is to maximize the mutual information between positive pairs while minimizing it for negative pairs. In this study, this means enhancing the representation of circRNAs and diseases that are truly associated while suppressing those that are not.The InfoNCE loss function is defined as follows:

$$L=-\sum_{i=1}^{N}\log\left(\frac{\sum_{j\in P_{i}}\exp\left(S_{ij}/\tau \right)}{\sum_{k\in N_{i}}\exp\left(S_{ik}/\tau \right)}\right),$$ (24)

where *N* represents the number of circRNAs, *P*_*i*_ and *N*_*i*_ are the positive and negative sample sets for the *i*-th circRNA, respectively, and τ is set to 0.1 by default.

### Evaluation metrics

Four evaluation criteria, Accuracy (ACC), Area Under the Receiver Operating Characteristic Curve (AUC), Area Under the Precision-Recall Curve (AUPR), and F1-score, are used to evaluate the performance of MVHGCN and benchmark methods. These criteria are widely used to evaluate machine learning models for classification because of their comprehensive reflection of accuracy, robustness, and stability. In the context of machine learning, *TP* represents the number of true positive samples, *FP* denotes the number of false positive samples, *TN* indicates the number of true negative samples, and *FN* stands for the number of false negative samples. AUC represents the area under a ROC curve plotted by the False Positive Rate (FPR) against the True Positive Rate (TPR). AUPR denotes the area under a precision-recall (PR) curve depicting the relationship between Precision (Pre) and Recall (Rec). These evaluation criteria are defined as:


$$\text{ACC}=\frac{TP+TN}{TP+TN+FP+FN}$$

$$\text{FPR}=\frac{FP}{FP+TN}$$

$$\text{TPR}=\frac{TP}{TP+FN}$$

$$\text{Pre}=\frac{TP}{TP+FP}$$

$$\text{Rec}=\frac{TP}{TP+FN}$$

$$\text{F1}=\frac{2TP}{2TP+FP+FN}$$

## Results

### Datasets

MVHGCN is evaluated on three heterogeneous datasets integrated from 10 databases to validate its effectiveness. These heterogeneous datasets are built by five different associations (circRNA-disease associations, circRNA-miRNA associations, lncRNA-disease associations, miRNA-disease associations, and lncRNA-miRNA associations). The primary distinction among the three datasets lies in the categorization of disease types: Dataset 1 includes only cancer-related diseases, Dataset 2 includes only non-cancer-related diseases, and Dataset 3 encompasses both cancer and non-cancer-related diseases. Specifically for Dataset 1, circRNA-disease associations are obtained from Circ2Disease [30], CircR2Disease [31], circR2Cancer [32], and Lnc2Cancer [33]. circRNA-miRNA associations are obtained from circR2Cancer and circBank [34]. lncRNA-disease associations are constructed from Lnc2Cancer and LncRNADisease. miRNA-disease associations are generated from circR2Cancer, Circ2Disease, HMDD [35], mir2disease [36], and miRCancer [37]. lncRNA-miRNA associations come from lncRNASNP2 [38]. Similarly, Dataset 2 and Dataset 3 are built using the same strategy. The difference from Dataset 1 lies in the source of the circRNA-disease associations: Dataset 2 obtains associations between circRNAs and non-cancer diseases from Circ2Disease, CircR2Disease, circR2Cancer, and Lnc2Cancer, while Dataset 3 derives its circRNA-disease associations from LncRNADisease [39]. The number of circRNA, diseases, miRNA, lncRNA, and their five associations is shown in Table 1.

**Table 1:** The number of circRNA, disease, miRNA, lncRNA, and their associations in the three datasets.

	Dataset1	Dataset2	Dataset3
circRNA	2480	1080	2640
disease	101	172	181
miRNA	2746	2716	2743
lncRNA	3291	3279	3383
circRNA-disease	3602	1273	3368
circRNA-miRNA	79908	50081	80015
miRNA-disease	735	780	315
lncRNA-disease	268	429	831
lncRNA-miRNA	31732	32073	33071

All three constructed datasets contain an equal number of positive and negative samples. Based on the assumption that similar circRNAs often have similar diseases, we use a stratified filtering method while constructing negative samples. Specifically, for a given circRNA *i*, we first determine whether other circRNAs shared the same associated diseases based on different association views. If multiple circRNAs share common disease associations with circRNA *i*, diseases not linked to these circRNAs are selected and paired with circRNA *i* to generate negative samples. However, constructing negative samples by the above method usually results in a small number of negative samples. Therefore, the negative sample construction strategy proposed by Wei *et al*. [12] is utilized to balance the number of positive and negative samples. Specifically, according to the circRNA similarity *CS*, *k* dissimilar circRNAs are selected to form a set: $CS_{i}=c_{1},c_{2},...,c_{k}$. Then all diseases associated with the circRNAs in *CS*_*i*_ are selected as a set: $CD_{i}=d_{1},d_{2},...,d_{n}$, where *n* is the number of associated diseases. Finally, diseases from *CD* are used to construct negative samples.

### Performance of MVHGCN on three datasets

**Fig 2:**
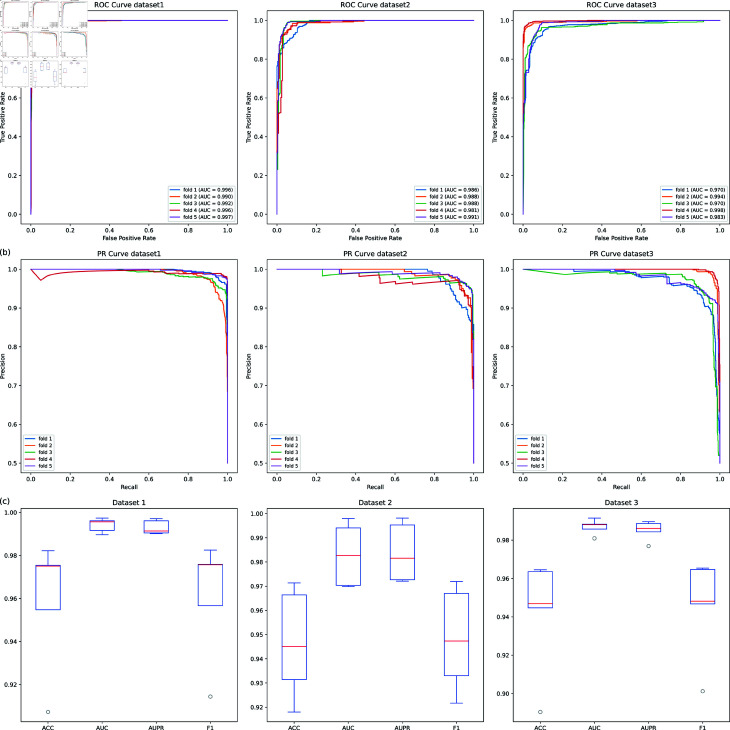
The ROC curves and results of MVHGCN obtained through 5-fold cross-validation on the three datasets. (a) ROC curves, (b) PR curves, (c) Box plots.

The prediction performance of MVHGCN is evalusted by 5-fold cross-validation on the three datasets, respectively. The ROC curves and PR curves obtained for each fold are shown in Fig 2. The ACC, AUC, AUPR, and F1-score of each fold are shown in Table 2. For the key metrics AUC and AUPR, MVHGCN exhibites relatively stable performance with high average values across three datasets, with an average AUC value of 99.42% and an average AUPR value of 99.28% on Dataset 1. However, for the metrics ACC and F1-score, the model showes fluctuations, which might be due to the limited data, as illustrated in Fig 2(c).

**Table 2:** The results obtained by MVHGCN through 5-fold cross-validation on the three datasets.

	Dataset	Fold 1	Fold 2	Fold 3	Fold 4	Fold 5	Average
ACC(%)	1	97.5	90.7	95.5	98.2	97.5	95.9±3.1
	2	89	94.5	96.4	94.7	96.5	94.2±3.1
	3	91.8	96.6	93.1	97.1	94.5	94.6±2.3
AUC(%)	1	99.6	99	99.2	99.6	99.7	99.4±0.3
	2	98.6	98.8	98.8	98.1	99.1	98.7±0.4
	3	97	99.4	97	99.8	98.3	98.3±1.3
AUPR(%)	1	99.6	99	99	99.1	99.7	99.3±0.3
	2	98.6	98.9	98.4	97.7	99	98.5±0.5
	3	97.2	99.5	97.3	99.8	98.2	98.4±1.2
F1-score(%)	1	97.6	91.4	95.7	98.3	97.6	96.1±2.8
	2	90.1	94.7	96.5	94.8	96.5	94.5±2.6
	3	92.2	96.7	93.3	97.2	94.7	94.8±2.1

### Comparison with six benchmark methods

**Fig 3:**
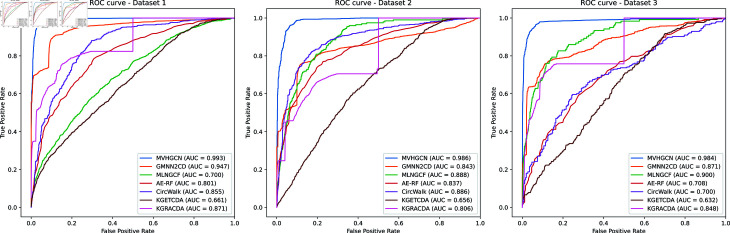
The ROC curves of MVHGCN compared with six benchmark methods on the three datasets.

To further validate the effectiveness of MVHGCN, it is compared with six benchmark methods, GMNN2CD [16], MLNGCF [19], AE-RF [20], CircWalk [21], KGETCDA [17], and KGRACDA [22]. AUC is selected as the primary comparison metric, and the ROC curves for each method are shown in Fig 3, with other metric values presented in Table 3. Across three datasets, MVHGCN achieves the highest AUC values of 0.993, 0.986, and 0.984, respectively, indicating that MVHGCN exhibits excellent generalization ability and classification performance across different datasets. The AUC values of the other benchmark methods are all significantly lower than those of MVHGCN, particularly on Dataset 1 and Dataset 2, where the performance gap is most pronounced. Although GMNN2CD showes relatively stable performance across all datasets, with AUC values around 0.881, it still does not perform as well as MVHGCN. MVHGCN achieves average ACC, AUPR, and F1-score values of 0.955, 0.987, and 0.955 across the three datasets, outperforming the second-best existing method by 0.109, 0.18, and 0.18, respectively. The performance of the other methods achieve relatively lower ACC, AUC, AUPR and F1-score values, further demonstrating the superior discriminative ability of MVHGCN in various datasets. This disparity highlights that MVHGCN not only possesses stronger robustness but also demonstrates the effectiveness of MVHGCN in alleviating data sparsity through multi-view feature extraction and improving performance via a reasonable negative sampling strategy.

**Table 3:** The results obtained by MVHGCN and six benchmark methods on the three datasets.

	Dataset	MVHGCN	GMNN2CD	MLNGCF	AE-RF	CircWalk	KGETCDA	KGRACDA
ACC(%)	1	96.8	89.6	64.2	73.9	79.2	60.0	80.2
	2	94.7	81.9	80.8	77.1	82.6	61.5	75.0
	3	94.9	82.4	82.5	65.8	67.5	60.9	80.0
	Average	95.5	84.6	75.8	72.3	76.4	60.8	78.4
AUC(%)	1	99.3	94.7	70.0	80.1	85.5	66.1	87.1
	2	98.6	84.3	88.8	83.7	88.6	65.6	80.6
	3	98.4	87.1	90.0	70.8	70.0	63.2	84.8
	Average	98.8	88.7	82.9	78.2	81.4	65.0	84.2
AUPR(%)	1	99.2	65.9	5.5	79.0	83.0	65.8	0.79
	2	98.4	36.9	7.0	84.7	87.8	65.5	0.34
	3	98.6	27.9	18.4	69.1	71.3	63.1	0.16
	Average	98.7	43.6	10.3	77.6	80.7	64.8	0.4
F1-score(%)	1	96.8	70.3	12.1	74.8	80.9	59.9	5.8
	2	94.8	48.4	13.4	77.6	82.7	61.4	3.89
	3	95.0	33.9	28.1	68.6	68.8	59.0	0.7
	Average	95.5	50.9	17.9	73.7	77.5	60.1	3.5

### Effectiveness of associated views in MVHGCN

**Fig 4:**
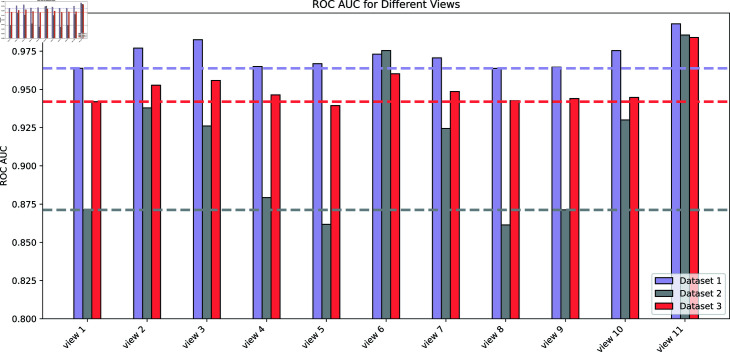
Performance comparison of different view construction methods on the three datasets.

To investigate the impact of different views on model prediction performance, 11 views are evaluated by 5-fold cross-validation on the three datasets, and the results are shown in Fig 4. These views were defined as follows: $view1=P_{cd}$, $view2=P_{cd}\;+\;P_{cmd}$, $view3=P_{cd}\;+\;P_{cdcd}$, $view4=P_{cd}\;+\;P_{cdld}$, $view5=P_{cd}\;+\;P_{cdmd}$, $view6=P_{cd}\;+\;P_{cmcd}$, $view7=P_{cd}\;+\;P_{cmld}$, $view8=P_{cd}\;+\;P_{cdlmd}$, $view9=P_{cd}\;+\;P_{cdmld}$, $view10=P_{cd}\;+\;P_{cmlmd}$. Taking Dataset1 as an example, the performance of view4, view5, view8, and view9 did not significantly improve after incorporating additional meta-path views on top of *P*_*cd*_, indicating that not all views enhance prediction performance. This may be due to the introduction of irrelevant information or noise by certain views. Ultimately, we select effective meta-path views to construct $view11=P_{cd}+P_{cmd}+P_{cdcd}+P_{cmcd}+P_{cmld}+P_{cmlmd}$. View11 demonstrated excellent performance on all three datasets.

### Performance comparison of different loss functions in MVHGCN

**Fig 5:**
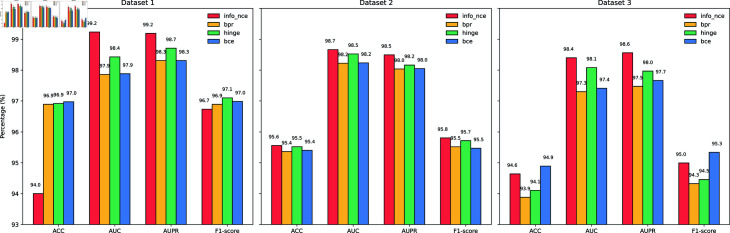
Performance comparison of different loss functions on the three datasets.

To evaluate the impact of loss functions on model performance, a 5-fold cross-validation is used on the three datasets, comparing InfoNCE with BPR [40], Hinge [41],and BCE [42]. The results are shown in Fig 5. InfoNCE demonstrates particularly better performance in terms of AUC and AUPR metrics, showcasing exceptional classification capabilities and the ability to distinguish between positive and negative samples, especially on Dataset1 and Dataset3. Although Hinge does not perform as well as InfoNCE, it still showes commendable results. BCE exhibits the most balanced performance on Dataset1, achieving the highest values for accuracy and F1-score. In contrast, BPR’s overall performance is inferior to that of the other loss functions, particularly on Dataset1 and Dataset3, where its accuracy and AUC show notable gaps. Overall, InfoNCE outperforms the other loss functions.

### Performance comparison of different GNNs in MVHGCN

**Fig 6:**
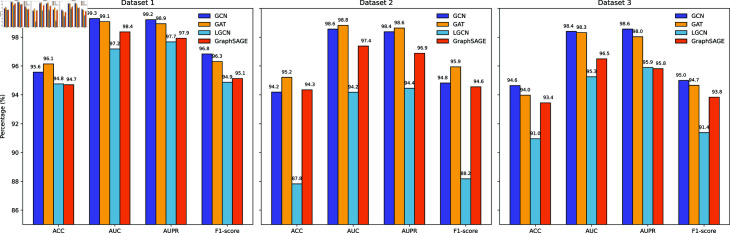
Performance comparison of different GNNs on the three datasets.

**Fig 7:**
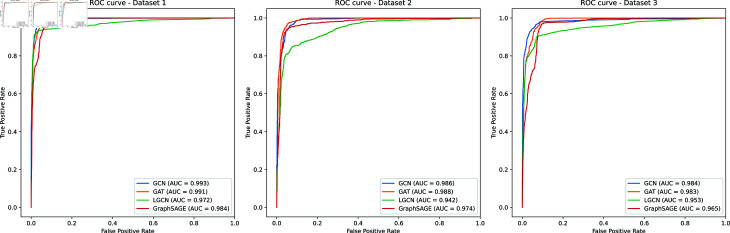
The ROC curves of MVHGCN with different GNNs on the three datasets.

To comprehensively evaluate the effectiveness of different Graph Neural Networks (GNNs), we replaces the GCN [28] in MVHGCN with GAT [43], LGCN [44], and GraphSAGE [45] respectively for comparison. Fig 6 presents the AUC results obtained through 5-fold cross-validation, and Fig 7 shows their ROC curves.

We observe that GCN and GAT exhibit similar performance, but because of GAT’s longer computational time, GCN is chosen as the final model. Compared to other models, GCN exhibits higher performance across all metrics. Taking Dataset 1 as an example, GCN achieves 0.8% higher ACC than LGCN and 0.9% higher than GraphSAGE. In terms of AUC, GCN demonstrates a 2.1% higher value than LGCN and 0.9% higher than GraphSAGE. For AUPR, GCN showes 1.5% higher values than LGCN and 1.3% higher than GraphSAGE. Finally, for the F1-score, GCN achieves 1.9% higher than LGCN and 1.7% higher than GraphSAGE.

### Parameter analysis

**Fig 8:**
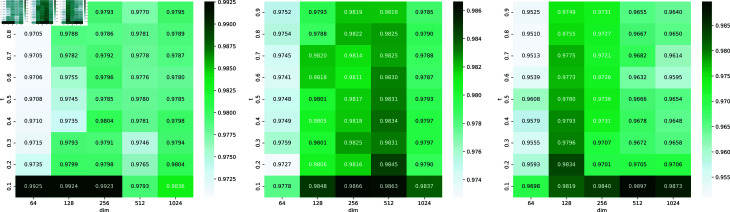
The impact of temperature and dimensionality on AUC values across the three datasets.

The impact of parameters in MVHGCN on performance are explored in this section, with a particular focus on the temperature parameter in InfoNCE and the setting of embedding dimensionality. The temperature parameter is used to adjust the sensitivity of similarity measurements in contrastive learning. We evaluate the effects of different temperatures (*t*) and dimensionality on the AUC value through 5-fold cross-validation on the three datasets, shown in Fig 8. Results show a slight performance decline as temperature (*t*) increases. For Dataset 1, performance improves significantly when dimensionality increases from 64 to 256 but plateaus or slightly declines beyond 512. Similar trends are observed in Dataset 2 and Dataset 3, where performance gains diminish at higher dimensionality. Overall, moderate dimensionality (256 or 512) achieves the best balance. Thus, the temperature is set to 0.1, and the embedding dimensionality is set to 256 in MVHGCN.

## Discussion

MVHGCN improves the accuracy by alleviating data sparsity and precisely selecting negative samples. Its methodology can be summarized into three key points: Firstly, MVHGCN employes a meta-path strategy to extract multiple relational views from heterogeneous graphs, capturing the diverse associations between circRNAs and diseases. This strategy not only enriches the associations between circRNAs and diseases but also improves the accuracy of negative sample selection. Secondly, by aggregating different views through graph convolutional networks, MVHGCN integrates various association patterns into more expressive embedding representations. Finally, in constructing and utilizing multiple associations between circRNAs and diseases, MVHGCN focuses on the interaction effects between different connection types on the target associations, ensuring that the model can effectively capture and express these complex interaction features.

To further validate the predictive performance of the model, we scored circRNA-disease pairs on Dataset 1 and confirmed the predictions by searching the literature in the PubMed database. Table 4 and Table 5 list the validation literature. Colorectal cancer (CRC) and hepatocellular carcinoma (HC), as malignant tumors with high global incidence and mortality rates, have been demonstrated in previous studies to involve circRNAs playing critical roles in their occurrence and progression [46,47]. Therefore, we selected colorectal cancer and hepatocellular carcinoma as validation targets for evaluating the predictive performance. The results showed that among the top 10 candidate circRNAs with the highest scores, 5 have been validated in the literature.

**Table 4:** Top 10 circRNAs highly associated with hepatocellular carcinoma predicted by MVHGCN.

Rank	circRNA	Disease	Evidence (PMID)
1	circFBXO11	HC	32222024
2	Circular RNA 101368	HC	30265210
3	circFNTA	HC	unknown
4	CircRNA FGFR3	HC	unknown
5	CircKIF5B	HC	35847962
6	circFLNA	HC	unknown
7	CircFMN2	HC	35909906
8	circFN1	HC	33230446
9	circFNDC3B	HC	unknown
10	VPS13C - has-circ-001567	HC	unknown

**Table 5:** Top 10 circRNAs highly associated with colorectal cancer predicted by MVHGCN.

Rank	circRNA	Disease	Evidence (PMID)
1	CircETFA	CRC	unknown
2	CircRNA-ENO1	CRC	36620599
3	circ-FARSA	CRC	32473899
4	CircEZH2	CRC	35773744
5	CircFADS2	CRC	32486849
6	CircFAM114A2	CRC	unknown
7	circFAM120A	CRC	unknown
8	circFAM169A	CRC	37616050
9	CircFAM73A	CRC	unknown
10	Circular RNA_0074027	CRC	33760126

Although the results are satisfactory, the method still has some limitations. On one hand, due to the inconsistent naming conventions, expression patterns, and descriptive focuses across different circRNA databases, it is still challenging to achieve uniformity, which affects the model’s generalization capability. On the other hand, the proposed method relies on known data to mine unknown associations and is unable to identify new associations between novel circRNAs and diseases. Additionally, although the method can uncover unknown associations, it lacks the ability to interpret their biological significance. Therefore, in future research, we will explore the use of representative circRNA databases and integrate RNA sequences, gene expression information, and functional roles to construct a regulatory network with greater biological meaning.

## Conclusion

CircRNAs have played a significant role in the diagnosis of various diseases, particularly in cancers, neurological disorders, and cardiovascular diseases. Accurately predicting the potential associations between circRNAs and diseases offers new perspectives for disease diagnosis and treatment strategies. However, existing methods have faced challenges in predicting circRNA-disease associations because of data sparsity and the difficulty in identifying negative samples. To address these issues, a novel prediction model, MVHGCN, is proposed based on multi-view and graph convolutional networks to predict potential circRNA-disease associations. Experimental results on the three datasets demonstrate that MHVGCN is significantly effective in predicting circRNA-disease associations, providing strong support for related disease research and clinical applications.
